# Supplementary material for: End-of-life targeted degradation of DAF-2 insulin/IGF-1 receptor promotes longevity free from growth-related pathologies
Source: eLife. 2021 Sep 10;10:e71335. doi: 10.7554/eLife.71335 (PMC8492056; doi:10.7554/eLife.71335)
Supplement: Supplementary file 1. — Trials that were performed in parallel are grouped together. (N) = number of animals observed; lifespan was measured from the L4 stage (see Materials and methods for details). Animals that left the plates, buried into the agar, bagged, or exploded were censored. L4440 empty vector was otherwise used as the control. The trial number is usually the starting date of the lifespan. All treatments were performed either starting from L4 or during adulthood, with DMSO and pL4440 empty vector plates used for rapamycin and RNAi controls, respectively. p-Values were obtained by the log-rank. [file elife-71335-supp1.docx]

**Supplementary Table 1. Lifespans of degron-tagged DAF-2**

| Strain / **RNAi** | Mean lifespan ± S.E.M.  [Days] | 75^th^ percentile  [Days] | N dead/ Initial N | % mean lifespan change to control | P-value (log-rank) vs. control |  |  |  |  |  |  |  |
| --- | --- | --- | --- | --- | --- | --- | --- | --- | --- | --- | --- | --- |
| Trial 1 of DAF-2::degron knock down with 1 mM Auxin starting at L4 stage at 20°C (with FUDR). | | | | | | |  |  |  |  |  |  |
| N2 DMSO (0.25%) | 16.4 ± 0.3 | 19 | 111/146 |  |  |  |  |  |  |  |  |  |
| N2 Auxin (1 mM) | 18.3 ± 0.4 | 23 | 126/127 | +11 | 7e-05 |  |  |  |  |  |  |  |
| *daf-2(e1368)* DMSO (0.25%) | 18.4 ± 0.7 | 23 | 98/103 | +12 | 4e-03 |  |  |  |  |  |  |  |
| *daf-2(e1368)* Auxin (1 mM) | 24.4 ± 0.6 | 30 | 114/119 | +48 | 3e-25 |  |  |  |  |  |  |  |
| DAF-2::degron DMSO (0.25%) | 17.1 ± 0.3 | 19 | 101/124 | +4 | 2e-01 |  |  |  |  |  |  |  |
| DAF-2::degron Auxin (1 mM) | 28.5 ± 0.6 | 48 | 105/107 | +74 | 6e-35 |  |  |  |  |  |  |  |
| *P*-value and % mean lifespan change are relative to N2 DMSO (0.25%) | | | | | | |  |  |  |  |  |  |
| Trial 2 of DAF-2::degron knock down with 1 mM Auxin starting at L4 stage at 20°C (with FUDR). | | | | | | |  |  |  |  |  |  |
| N2 Auxin (1 mM) | 18.9 ± 0.6 | 23 | 42/82 |  |  |  |  |  |  |  |  |  |
| *daf-2(e1368)* Auxin (1 mM) | 22.3 ± 0.6 | 25 | 62/101 | +18 | 1e-03 |  |  |  |  |  |  |  |
| *daf-2(e1370)* DMSO (0.25%) | 33.6 ± 1 | 40 | 103/115 | +77 | 3e-23 |  |  |  |  |  |  |  |
| *daf-2(e1370)* Auxin (1 mM) | 32.1 ± 0.9 | 40 | 106/112 | +70 | 5e-20 |  |  |  |  |  |  |  |
| DAF-2::degron Auxin (1 mM) | 36.6 ± 1.3 | 47 | 91/93 | +93 | 5e-20 |  |  |  |  |  |  |  |
| DAF-2::degron Auxin (Young) (1 mM)* | 35.3± 1.8 | 48 | 63/72 | +87 | 1e-11 |  |  |  |  |  |  |  |
| ***Animals that evaded dauer entry (see fig. ). *P*-value and % mean lifespan change are relative to N2 Auxin (1 mM) | | | | | | |  |  |  |  |  |  |
| Trial 3 of DAF-2::degron knock down with 1 mM Auxin starting at L4 stage at 20°C (with FUDR). | | | | | | |  |  |  |  |  | mb |
| N2 DMSO (0.25%) | 15.3 ± 0.5 | 18 | 50/82 |  |  |  |  |  |  |  |  |  |
| N2 Auxin (1 mM) | 15.6 ± 0.4 | 18 | 64/98 | +2 | 6e-01 |  |  |  |  |  |  |  |
| *daf-2(e1368)* Auxin (1 mM) | 24.6 ± 0.8 | 30 | 66/85 | +61 | 3e-15 |  |  |  |  |  |  |  |
| *daf-2(e1370)* DMSO (0.25%) | 32.5 ± 1.0 | 40 | 83/93 | +113 | 9e-27 |  |  |  |  |  |  |  |
| *daf-2(e1370)* Auxin (1 mM) | 32.7 ± 0.9 | 42 | 99/105 | +114 | 8e-32 |  |  |  |  |  |  |  |
| DAF-2::degron DMSO (0.25%) | 15.1 ± 0.4 | 18 | 66/85 | -1 | 8e-0.1 |  |  |  |  |  |  |  |
| DAF-2::degron Auxin (1 mM) | 35.8 ± 1.3 | 55 | 63/67 | +134 | 2e-24 |  |  |  |  |  |  |  |
| *P*-value and % mean lifespan change are relative to wild type (N2) DMSO | | | | | | |  |  |  |  |  |  |
| Trial 4 of DAF-2::degron knock down with 1 mM Auxin starting at L4 stage at 20°C (with FUDR). | | | | | | |  |  |  |  |  | ce; 1h |
| N2 DMSO (0.25%) | 18.9 ± 0.3 | 22 | 274/281 |  |  |  |  |  |  |  |  |  |
| N2 Auxin (1 mM) | 20.5 ± 0.3 | 24 | 278/280 | +9 | 2e-05 |  |  |  |  |  |  |  |
| *daf-2(e1368)* DMSO (0.25%) | 30.4 ± 0.6 | 36 | 226/229 | +61 | 5e-61 |  |  |  |  |  |  |  |
| *daf-2(e1368)* Auxin (1 mM) | 29.6 ± 0.5 | 36 | 255/257 | +57 | 4e-69 |  |  |  |  |  |  |  |
| *daf-2(e1370)* DMSO (0.25%) | 35.6 ± 0.5 | 42 | 175/178 | +89 | 6e-75 |  |  |  |  |  |  |  |
| *daf-2(e1370)* Auxin (1 mM) | 36.4 ± 0.6 | 44 | 214/216 | +94 | 8e-91 |  |  |  |  |  |  |  |
| DAF-2::degron DMSO (0.25%) | 19.1 ± 0.3 | 24 | 303/315 | +1 | 4e-01 |  |  |  |  |  |  |  |
| DAF-2::degron Auxin (1 mM) | 38.3 ± 0.5 | 48 | 269/270 | +103 | 3e-111 |  |  |  |  |  |  |  |
| *P*-value and % mean lifespan change are relative to wild type (N2) DMSO | | | | | | |  |  |  |  |  |  |
| **Merge** of all 4 trials of DAF-2::degron knock down with 1 mM Auxin starting at L4 stage at 20°C (with FUDR). | | | | | | |  |  |  |  |  | kp |
| N2 DMSO (0.25%) | 17.8 ± 0.2 | 20 | 435/479 |  |  |  |  |  |  |  |  |  |
| N2 Auxin (1 mM) | 19.2 ± 0.2 | 23 | 510/587 | +8 | 2e-06 |  |  |  |  |  |  |  |
| *daf-2(e1368)* DMSO (0.25%) | 26.8 ± 0.5 | 34 | 324/332 | +51 | 5e-57 |  |  |  |  |  |  |  |
| *daf-2(e1368)* Auxin (1 mM) | 27.1 ± 0.3 | 32 | 497/562 | +52 | 1e-99 |  |  |  |  |  |  |  |
| *daf-2(e1370)* DMSO (0.25%) | 34.1 ± 0.5 | 42 | 361/386 | +92 | 1e-140 |  |  |  |  |  |  |  |
| *daf-2(e1370)* Auxin (1 mM) | 34.4 ± 0.4 | 42 | 419/433 | +93 | 5e-158 |  |  |  |  |  |  |  |
| DAF-2::degron DMSO (0.25%) | 18.1 ± 0.2 | 22 | 469/527 | +2 | 2e-01 |  |  |  |  |  |  |  |
| DAF-2::degron Auxin (1 mM) | 36.7 ± 0.4 | 50 | 528/537 | +107 | 8e-188 |  |  |  |  |  |  |  |
| *P*-value and % mean lifespan change are relative to wild type (N2) DMSO | | | | | | |  |  |  |  |  |  |
| P-Value matrix for **Merge** of all 4 trials   \|  \| N2  DMSO (0.25%) \| N2  Auxin (1 mM) \| *daf-2(e1368)* DMSO (0.25%) \| *daf-2(e1368)* Auxin (1 mM) \| DAF-2::degron DMSO (0.25%) \| DAF-2::degron Auxin (1 mM) \| *daf-2(e1370)* DMSO (0.25%) \| \| --- \| --- \| --- \| --- \| --- \| --- \| --- \| --- \| \| N2  Auxin (1 mM) \| <0.0001 \|  \|  \|  \|  \|  \|  \| \| *daf-2(e1368)* DMSO (0.25%) \| <0.0001 \| <0.0001 \|  \|  \|  \|  \|  \| \| *daf-2(e1368)* Auxin (1 mM) \| <0.0001 \| <0.0001 \| n.s (0.098) \|  \|  \|  \|  \| \| DAF-2::degron DMSO (0.25%) \| n.s (0.214) \| <0.0001 \| <0.0001 \| <0.0001 \|  \|  \|  \| \| DAF-2::degron Auxin (1 mM) \| <0.0001 \| <0.0001 \| <0.0001 \| <0.0001 \| <0.0001 \|  \|  \| \| *daf-2(e1370)* DMSO (0.25%) \| <0.0001 \| <0.0001 \| <0.0001 \| <0.0001 \| <0.0001 \| <0.0001 \|  \| \| *daf-2(e1370)* Auxin (1 mM) \| <0.0001 \| <0.0001 \| <0.0001 \| <0.0001 \| <0.0001 \| <0.0001 \| n.s (0.557) \|   Trial 1 of tissue-specific knockdown of degron-tagged DAF-2 with 0.1 mM starting at L4 stage at 20°C (with FuDR). | | | | | | |  |  |  |  |  | 1g |
| DAF-2::degron DMSO (0.025%) | 20.2 ± 0.4 | 23 | 106/118 |  |  |  |  |  |  |  |  |  |
| DAF-2::degron Auxin (0.1 mM) | 37.8 ± 0.8 | 53 | 109/109 | +87 | 6e-41 |  |  |  |  |  |  |  |
| Neuronal TIR1; *daf-2(bch40)* DMSO (0.025%) | 19.2 ± 0.8 | 25 | 80/84 | -5 | 9e-01 |  |  |  |  |  |  |  |
| Neuronal TIR1; *daf-2(bch40)* Auxin (0.1 mM) | 33.3 ± 0.9 | 40 | 97/98 | +65 | 2e-28 |  |  |  |  |  |  |  |
| Muscular TIR1; *daf-2(bch40)* Auxin (0.1 mM) | 16.7 ± 0.3 | 20 | 86/106 | -18 | 2e-07 |  |  |  |  |  |  |  |
| Intestinal TIR1; *daf-2(bch40)* Auxin (0.1 mM) | 27.7 ± 0.3 | 42 | 102/113 | 37 | 2e-09 |  |  |  |  |  |  |  |
| *P*-value and % mean lifespan change are relative to DAF-2::degron DMSO (0.025%) | | | | | | |  |  |  |  |  |  |
| Trial 2 of tissue-specific knockdown of degron-tagged DAF-2 with 0.1 mM starting at L4 stage at 20°C (with FuDR). | | | | | | |  |  |  |  |  | ce |
| DAF-2::degron DMSO (0.025%) | 16.5 ± 0.4 | 19 | 80/110 |  |  |  |  |  |  |  |  |  |
| DAF-2::degron Auxin (0.1 mM) | 31.3 ± 0.6 | 40 | 96/100 | +90 | 2e-37 |  |  |  |  |  |  |  |
| Neuronal TIR1; *daf-2(bch40)* DMSO (0.025%) | 21.8 ± 0.7 | 28 | 92/123 | +32 | 3e-10 |  |  |  |  |  |  |  |
| Neuronal TIR1; *daf-2(bch40)* Auxin (0.1 mM) | 27.4 ± 0.6 | 34 | 107/121 | +66 | 2e-30 |  |  |  |  |  |  |  |
| Muscular TIR1; *daf-2(bch40)* DMSO (0.025%) | 19.7 ± 0.5 | 23 | 99/114 | +20 | 6e-06 |  |  |  |  |  |  |  |
| Muscular TIR1; *daf-2(bch40)* Auxin (0.1 mM) | 20.1 ± 0.7 | 26 | 92/107 | +22 | 2e-05 |  |  |  |  |  |  |  |
| Intestinal TIR1; *daf-2(bch40)* DMSO (0.025%) | 18.8 ± 0.6 | 21 | 74/92 | +14 | 1e-03 |  |  |  |  |  |  |  |
| Intestinal TIR1; *daf-2(bch40)* Auxin (0.1 mM) | 29.0 ± 0.6 | 40 | 116/133 | +75 | 3e-33 |  |  |  |  |  |  |  |
| *P*-value and % mean lifespan change are relative to DAF-2::degron DMSO (0.025%) | | | | | | |  |  |  |  |  |  |
| Trial 3 of tissue-specific knockdown of degron-tagged DAF-2 with 0.1 mM starting at L4 stage at 20°C (with FuDR). | | | | | | |  |  |  |  |  | lm |
| DAF-2::degron DMSO (0.025%) | 19.3 ± 0.4 | 25 | 146/153 |  |  |  |  |  |  |  |  |  |
| DAF-2::degron Auxin (0.1 mM) | 29.5 ± 0.6 | 52 | 104/105 | +53 | 8e-30 |  |  |  |  |  |  |  |
| Neuronal TIR1; *daf-2(bch40)* DMSO (0.025%) | 17.9 ± 0.6 | 22 | 111/112 | -7 | 3e-01 |  |  |  |  |  |  |  |
| Neuronal TIR1; *daf-2(bch40)* Auxin (0.1 mM) | 24.3 ± 0.7 | 33 | 154/154 | +26 | 1e-12 |  |  |  |  |  |  |  |
| Muscular TIR1; *daf-2(bch40)* DMSO (0.025%) | 15.4 ± 0.5 | 19 | 100/100 | -20 | 6e-07 |  |  |  |  |  |  |  |
| Muscular TIR1; *daf-2(bch40)* Auxin (0.1 mM) | 15.2 ± 0.6 | 17 | 105/105 | -21 | 3e-05 |  |  |  |  |  |  |  |
| Intestinal TIR1; *daf-2(bch40)* DMSO (0.025%) | 16.7 ± 0.7 | 22 | 95/95 | -14 | 3e-02 |  |  |  |  |  |  |  |
| Intestinal TIR1; *daf-2(bch40)* Auxin (0.1 mM) | 23.2 ± 0.9 | 35 | 88/88 | +20 | 4e-07 |  |  |  |  |  |  |  |
| *P*-value and % mean lifespan change are relative to DAF-2::degron DMSO (0.025%) | | | | | | |  |  |  |  |  |  |
| **Merge** of 3 Trials of tissue-specific knockdown of degron-tagged DAF-2 with 0.1 mM starting at L4 stage at 20°C (with FuDR). | | | | | | |  |  |  |  |  | ce |
| DAF-2::degron DMSO (0.025%) | 18.9 ± 0.3 | 22 | 332/381 |  |  |  |  |  |  |  |  |  |
| DAF-2::degron Auxin (0.1 mM) | 31.8 ± 0.4 | 50 | 309/314 | +68 | 1e-105 |  |  |  |  |  |  |  |
| Neuronal TIR1; *daf-2(bch40)* DMSO (0.025%) | 19.6 ± 0.4 | 25 | 283/319 | +4 | 3e-03 |  |  |  |  |  |  |  |
| Neuronal TIR1; *daf-2(bch40)* Auxin (0.1 mM) | 27.2 ± 0.4 | 35 | 358/373 | +44 | 2e-58 |  |  |  |  |  |  |  |
| Muscular TIR1; *daf-2(bch40)* DMSO (0.025%) | 17.6 ± 0.4 | 21 | 199/214 | -7 | 1e-01 |  |  |  |  |  |  |  |
| Muscular TIR1; *daf-2(bch40)* Auxin (0.1 mM) | 17.4 ± 0.4 | 21 | 283/318 | -8 | 5e-02 |  |  |  |  |  |  |  |
| Intestinal TIR1; *daf-2(bch40)* DMSO (0.025%) | 17.7 ± 0.5 | 22 | 169/187 | -6 | 2e-01 |  |  |  |  |  |  |  |
| Intestinal TIR1; *daf-2(bch40)* Auxin (0.1 mM) | 26.5 ± 0.5 | 38 | 306/334 | +40 | 3e-43 |  |  |  |  |  |  |  |
| *P*-value and % mean lifespan change are relative to DAF-2::degron DMSO (0.025%) | | | | | | |  |  |  |  |  |  |
| P-Value matrix for **Merge** of all 3 trials   \|  \| DAF-2::degron DMSO \| DAF-2::degron  Auxin \| Neuronal TIR1; *daf-2(bch40) DMSO* \| Neuronal TIR1; *daf-2(bch40)* Auxin \| Muscular TIR1; *daf-2(bch40)* DMSO \| Muscular TIR1; *daf-2(bch40)* Auxin \| Intestinal TIR1; *daf-2(bch40)* DMSO \| \| --- \| --- \| --- \| --- \| --- \| --- \| --- \| --- \| \| DAF-2::degron  Auxin \| <0.0001 \|  \|  \|  \|  \|  \|  \| \| Neuronal TIR1; *daf-2(bch40) DMSO* \| 0.003 \| <0.0001 \|  \|  \|  \|  \|  \| \| Neuronal TIR1; *daf-2(bch40)* Auxin \| <0.0001 \| <0.0001 \| <0.0001 \|  \|  \|  \|  \| \| Muscular TIR1; *daf-2(bch40)* DMSO \| n.s (0.11) \| <0.0001 \| <0.0001 \| <0.0001 \|  \|  \|  \| \| Muscular TIR1; *daf-2(bch40)* Auxin \| n.s (0.54) \| <0.0001 \| <0.0001 \| <0.0001 \| n.s (0.933) \|  \|  \| \| Intestinal TIR1; *daf-2(bch40)* DMSO \| n.s (0.291) \| <0.0001 \| 0.002 \| <0.0001 \| n.s (0.87) \| n.s (0.902) \|  \| \| Intestinal TIR1; *daf-2(bch40)* Auxin \| <0.0001 \| <0.0001 \| <0.0001 \| 0.01 \| <0.0001 \| <0.0001 \| <0.0001 \| | | | | | | |  |  |  |  |  | lm |
| Trial of DAF-2::degron knock down with **0.1 mM** Auxin starting at L4 stage at 20°C (with FUDR). | | | | | | |  |  |  |  |  | ce ED3b |
| N2 DMSO (0.025%) | 14.7 ± 0.2 | 16 | 211/262 |  |  |  |  |  |  |  |  |  |
| N2 Auxin (0.1 mM) | 17.2 ± 0.2 | 20 | 262/268 | +17 | 6e-19 |  |  |  |  |  |  |  |
| *daf-2(e1368)* DMSO (0.025%) | 19.6 ± 0.3 | 22 | 340/358 | +33 | 1e-47 |  |  |  |  |  |  |  |
| *daf-2(e1368)* Auxin (0.1 mM) | 23.9 ± 0.4 | 30 | 309/325 | +63 | 4e-78 |  |  |  |  |  |  |  |
| *daf-2(e1370)* DMSO (0.025%) | 29.7 ± 0.4 | 40 | 228/229 | +102 | 6e-100 |  |  |  |  |  |  |  |
| *daf-2(e1370)* Auxin (0.1 mM) | 30.3 ± 0.4 | 44 | 237/240 | +106 | 6e-100 |  |  |  |  |  |  |  |
| DAF-2::degron DMSO (0.025%) | 15.0 ± 0.1 | 16 | 324/341 | +2 | 3e-01 |  |  |  |  |  |  |  |
| DAF-2::degron Auxin (0.1 mM) | 31.3 ± 0.4 | 40 | 242/251 | +113 | 9e-114 |  |  |  |  |  |  |  |
|  |  |  |  |  |  |  |  |  |  |  |  |  |
|  |  |  |  |  |  |  |  |  |  |  |  |  |
| *P*-value and % mean lifespan change are relative to wild type (N2) DMSO | | | | | | |  |  |  |  |  |  |
| Trial 1 of late life auxin treatment with 1 mM Auxin at 20°C (with FuDR). Strain = DAF-2::degron. | | | | | | |  |  |  |  |  | rv, ED3a |
| DMSO (0.25%) | 18.7 ± 0.2 | 20 | 221/386 |  |  |  |  |  |  |  |  |  |
| Auxin at L4 ( = day 0) | 43.4 ± 0.6 | 55 | 354/355 | +132 | 6e-127 |  |  |  |  |  |  |  |
| Auxin on day 3 | 37.5 ± 0.6 | 45 | 235/247 | +100 | 8e-83 |  |  |  |  |  |  |  |
| Auxin on day 7 | 36.7 ± 0.5 | 42 | 294/348 | +96 | 4e-94 |  |  |  |  |  |  |  |
| Auxin on day 10 | 32.2 ± 0.6 | 37 | 179/321 | +72 | 7e-68 |  |  |  |  |  |  |  |
| Auxin on day 12 | 28 ± 0.6 | 34 | 148/211 | +49 | 3e-4’ |  |  |  |  |  |  |  |
| *P*-value and % mean lifespan change are relative to DMSO | | | | | | |  |  |  |  |  |  |
| Trial 2 of late life auxin treatment with 1 mM Auxin at 20°C (with FuDR). Strain = DAF-2::degron. | | | | | | |  |  |  |  |  | rv, ED3d |
| DMSO (0.25%) | 21.9 ± 0.3 | 25 | 283/291 |  |  |  |  |  |  |  |  |  |
| Auxin at L4 ( = day 0) | 41.7 ± 0.7 | 54 | 292/300 | +91 | 4e-89 |  |  |  |  |  |  |  |
| Auxin on day 7 | 34.1 ± 1 | 47 | 164/166 | +56 | 3e-33 |  |  |  |  |  |  |  |
| Auxin on day 10 | 32.3 ± 1.1 | 44 | 139/145 | +48 | 1e-26 |  |  |  |  |  |  |  |
| Auxin on day 12 | 34.4 ± 1.1 | 44 | 134/142 | +57 | 2e-37 |  |  |  |  |  |  |  |
| Auxin on day 14 | 23.7 ± 0.8 | 30 | 155/156 | +8 | 4e-05 |  |  |  |  |  |  |  |
| Auxin on day 16 | 26.3 ± 0.8 | 32 | 140/142 | +21 | 3e-12 |  |  |  |  |  |  |  |
| Auxin on day 18 | 25.7 ± 0.7 | 32 | 156/163 | +18 | 1e-11 |  |  |  |  |  |  |  |
| Auxin on day 20 | 25.6 ± 0.7 | 32 | 146/150 | +17 | 3e-12 |  |  |  |  |  |  |  |
| *P*-value and % mean lifespan change are relative to DMSO | | | | | | |  |  |  |  |  |  |
| Trial 3 of late life auxin treatment with 1 mM Auxin at 20°C (with FuDR). Strain = DAF-2::degron. | | | | | | |  |  |  |  |  | rv |
| DMSO (0.25%) | 18.5 ± 0.3 | 16 | 102/116 |  |  |  |  |  |  |  |  |  |
| Auxin on day 7 | 29 ± 0.8 | 21 | 98/124 | +56 | 1e-23 |  |  |  |  |  |  |  |
| Auxin on day 10 | 29.4 ± 0.8 | 21 | 106/118 | +59 | 9e-25 |  |  |  |  |  |  |  |
| Auxin on day 16 | 18.9 ± 0.5 | 16 | 99/113 | +2 | 6e-01 |  |  |  |  |  |  |  |
| Auxin on day 18 | 19.1 ± 0.4 | 16 | 106/118 | +3 | 3e-01 |  |  |  |  |  |  |  |
| *P*-value and % mean lifespan change are relative to DMSO | | | | | | |  |  |  |  |  |  |
| Trial 1 *skn-1* RNAi with 0.1 mM Auxin at **15°C** (with FUDR). Strain = DAF-2::degron | | | | | | |  |  |  |  |  | rv |
| L4440 | 59.2 ± 2.2 | 77 | 79/79 |  |  |  |  |  |  |  |  |  |
| *skn-1* | 36.7 ± 1.1 | 43 | 91/93 | -38 | 1e-18 |  |  |  |  |  |  |  |
| *P*-value and % mean lifespan change are relative to L4440 | | | | | | |  |  |  |  |  |  |
| Trial 2 *skn-1* RNAi with 0.1 mM Auxin at **15°C** (with FUDR). Strain = DAF-2::degron | | | | | | |  |  |  |  |  | ce; 4a |
| L4440 | 58.8 ± 2 | 75 | 89/96 |  |  |  |  |  |  |  |  |  |
| *skn-1* | 42.1 ± 1.3 | 53 | 79/81 | -28 | 6e-13 |  |  |  |  |  |  |  |
| *P*-value and % mean lifespan change are relative to L4440 | | | | | | |  |  |  |  |  |  |
| **Merge** of *skn-1* RNAi with 0.1 mM Auxin at **15°C** (with FUDR). Strain = DAF-2::degron | | | | | | |  |  |  |  |  | ce |
| L4440 | 60 ± 1.6 | 77 | 168/175 |  |  |  |  |  |  |  |  |  |
| *skn-1* | 39.2 ± 0.8 | 28 | 158/181 | -35 | 1e-30 |  |  |  |  |  |  |  |
| *P*-value and % mean lifespan change are relative to L4440 | | | | | | |  |  |  |  |  |  |
| Trial 1 *skn-1* RNAi with 0.1 mM Auxin at **25°C** (with FUDR). Strain = DAF-2::degron | | | | | | |  |  |  |  |  | ce; |
| L4440 | 15.1 ± 0.6 | 27 | 77/78 |  |  |  |  |  |  |  |  |  |
| *skn-1* | 20.4 ± 0.7 | 32 | 104/105 | +35 | 6e-08 |  |  |  |  |  |  |  |
| *P*-value and % mean lifespan change are relative to L4440 | | | | | | |  |  |  |  |  |  |
| Trial 2 *skn-1* RNAi with 0.1 mM Auxin at **25°C** (with FUDR). Strain = DAF-2::degron | | | | | | |  |  |  |  |  | cs; ED3h |
| L4440 | 16.6 ± 0.7 | 20 | 69/80 |  |  |  |  |  |  |  |  |  |
| *skn-1* | 19.4 ± 0.7 | 26 | 90/101 | +17 | 1e-02 |  |  |  |  |  |  |  |
| *P*-value and % mean lifespan change are relative to L4440 | | | | | | |  |  |  |  |  |  |
| **Merge** of *skn-1* RNAi with 0.1 mM Auxin at **25°C** (with FUDR). Strain = DAF-2::degron | | | | | | |  |  |  |  |  | co |
| L4440 | 15.8 ± 0.5 | 25 | 146/158 |  |  |  |  |  |  |  |  |  |
| *skn-1* | 20 ± 0.5 | 21 | 194/206 | +26 | 5e-08 |  |  |  |  |  |  |  |
| *P*-value and % mean lifespan change are relative to wild type (N2) | | | | | | |  |  |  |  |  |  |
| Trial 1 of late life top-coating with auxin for a final concentration of 1 mM of auxin at 20°C (with FuDR). Strain = DAF-2::degron. (20200831) | | | | | | |  |  |  |  |  | rv |
| DMSO (0.25%) on day 10 | 19.5 ± 0.2 | 21 | 204/232 |  |  |  |  |  |  |  |  |  |
| Auxin on day 10 | 26.8 ± 1.0 | 32 | 64/81 | +37 | <0.0001 |  |  |  |  |  |  |  |
| Auxin on day 21 | 21.2 ± 0.4 | 23 | 213/247 | +8 | 0.0001 |  |  |  |  |  |  |  |
| *P*-value and % mean lifespan change are relative to DMSO | | | | | | |  |  |  |  |  |  |
| Trial 2 of late life top-coating with auxin for a final concentration of 1 mM of auxin at 20°C (with FuDR). Strain = DAF-2::degron. (20200911) | | | | | | |  |  |  |  |  | rv |
| DMSO (0.25%) on day 20 | 23.2 ± 0.5 | 26 | 113/131 |  |  |  |  |  |  |  |  |  |
| Auxin on day 20 | 29.6 ± 1.1 | 38 | 110/130 | +28 | <0.0001 |  |  |  |  |  |  |  |
| Auxin on day 24 | 26.9 ± 0.8 | 31 | 155/179 | +16 | <0.0001 |  |  |  |  |  |  |  |
| *P*-value and % mean lifespan change are relative to DMSO | | | | | | |  |  |  |  |  |  |
| Trial 4 of late life top-coating with auxin for a final concentration of 1 mM of auxin at 20°C (with FuDR). Strain = DAF-2::degron. (20200908) | | | | | | |  |  |  |  |  | rv |
| DMSO (0.25%) on day 20 | 22.3 ± 0.5 | 27 | 97/103 |  |  |  |  |  |  |  |  |  |
| Auxin on day 20 | 30.6 ± 1.0 | 41 | 124/136 | +37 | <0.0001 |  |  |  |  |  |  |  |
| Auxin on day 24 | 26.8 ± 0.8 | 31 | 174/193 | +20 | <0.0001 |  |  |  |  |  |  |  |
| *P*-value and % mean lifespan change are relative to DMSO | | | | | | |  |  |  |  |  |  |
