# Supplementary material for: End-of-life targeted degradation of DAF-2 insulin/IGF-1 receptor promotes longevity free from growth-related pathologies
Source: eLife. 2021 Sep 10;10:e71335. doi: 10.7554/eLife.71335 (PMC8492056; doi:10.7554/eLife.71335)

A

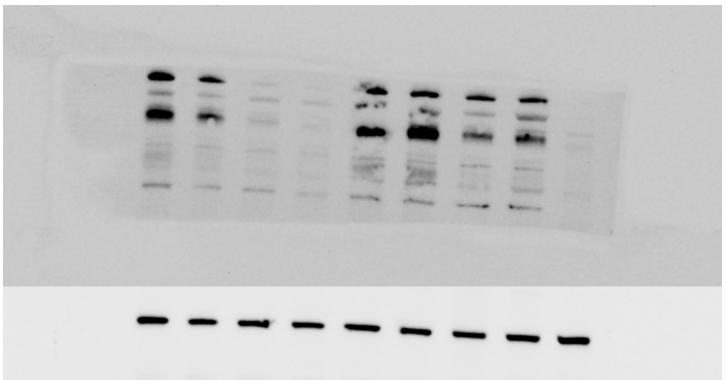

B

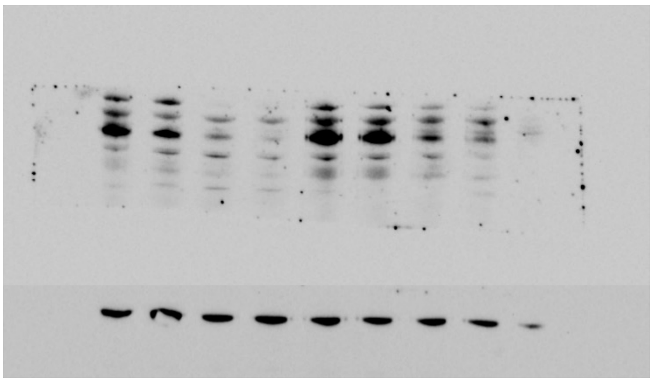

C

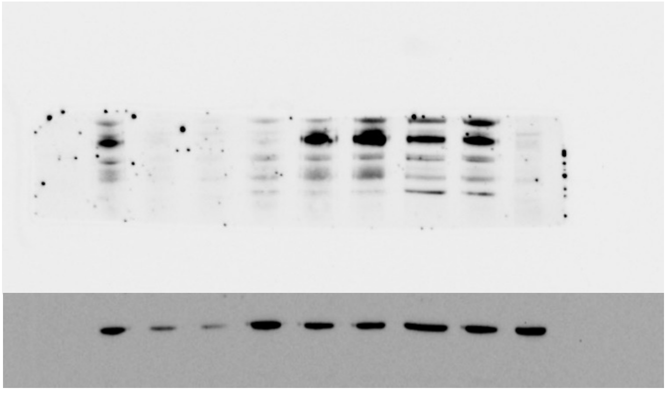

D

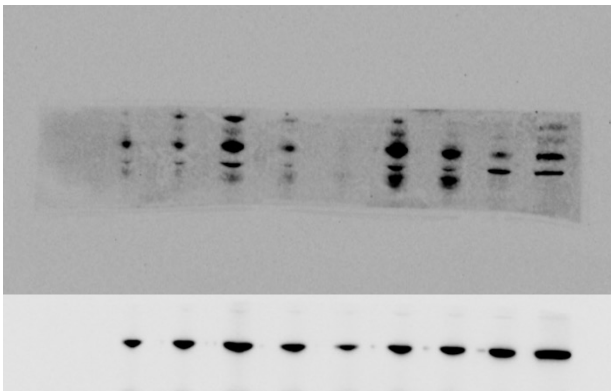

E

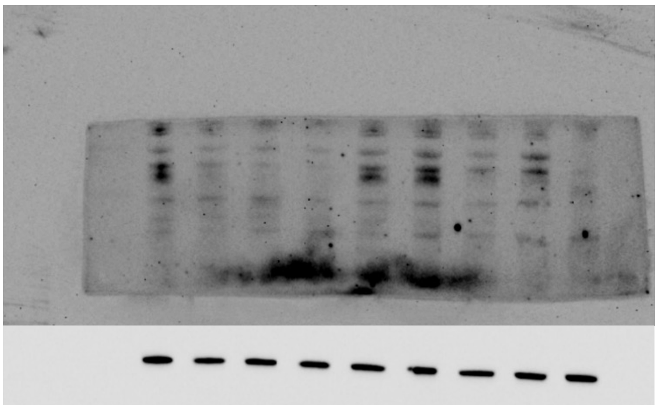

F

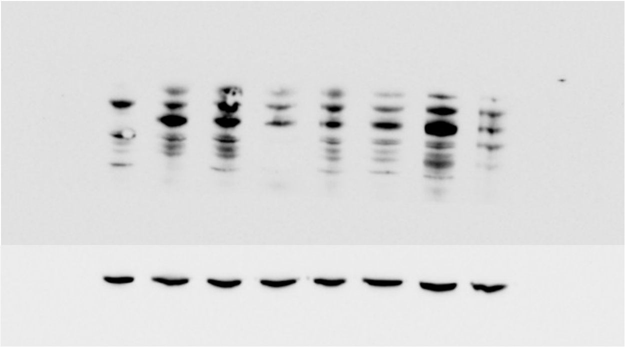

G

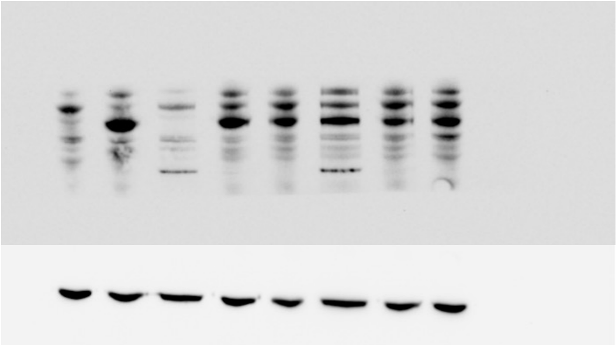

I 25.7

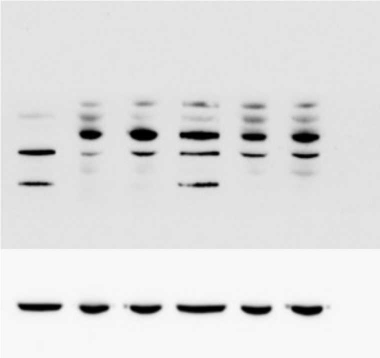

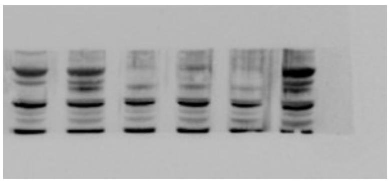

25.7

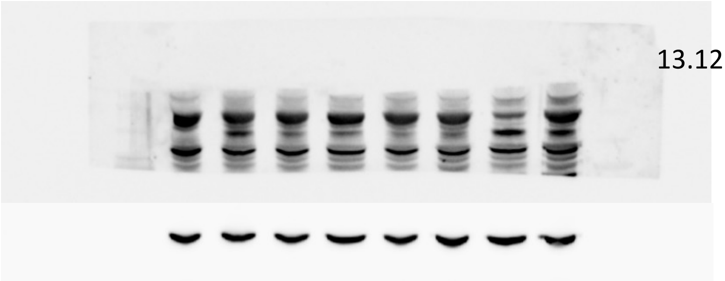

13.12

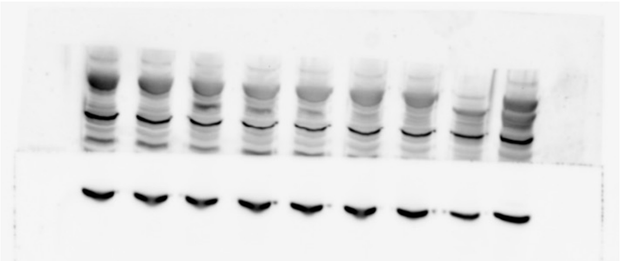

2.1

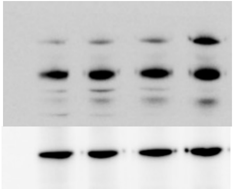

A*

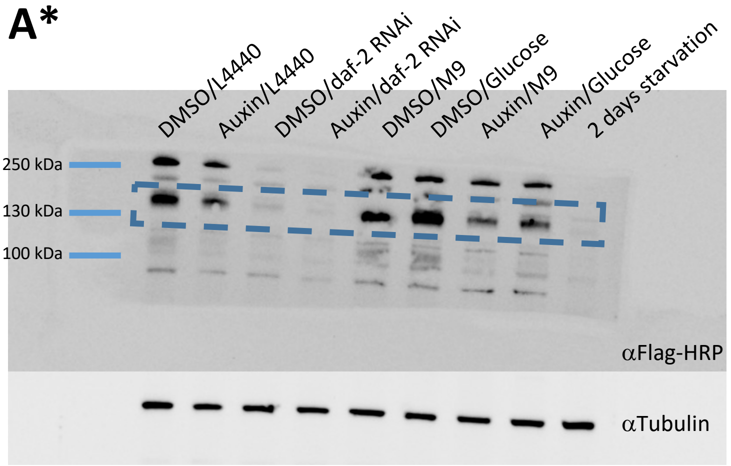

(This blot was used for fig. 1 C and 1F)

B*

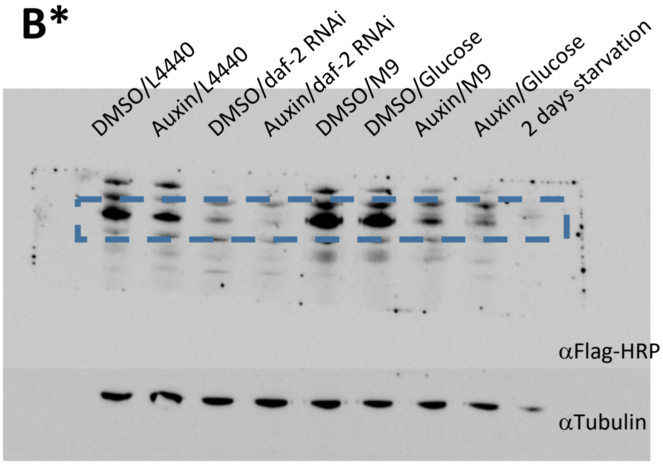

C*

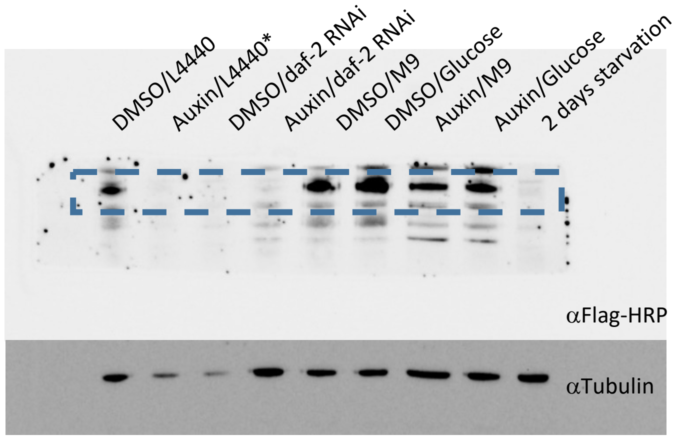

*: sample was sligthly starved when harvested and therefore excluded from analysis

D*

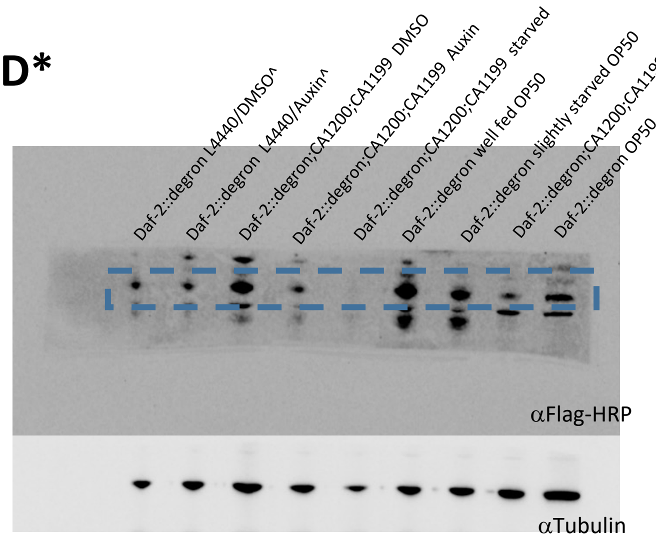

^: used for quantification in figure 1 I

E*

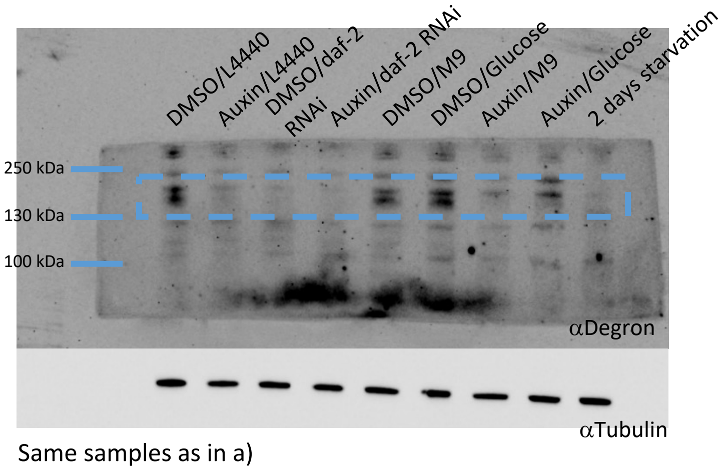

Same samples as in a)

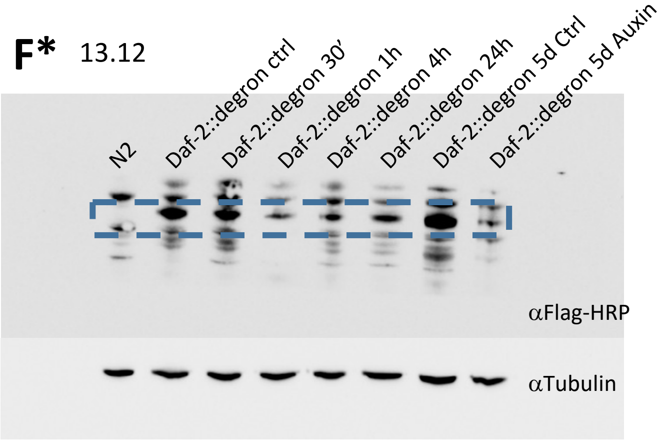

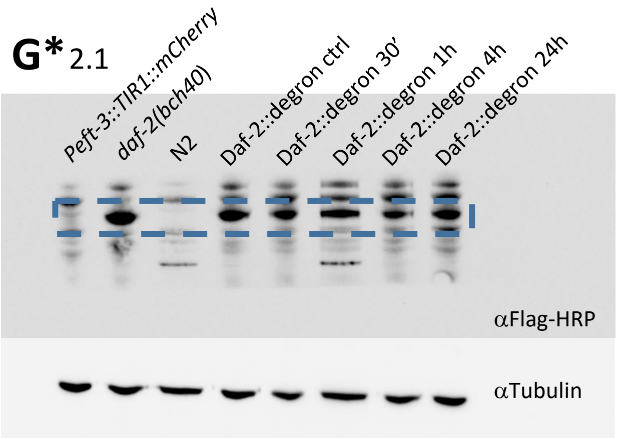

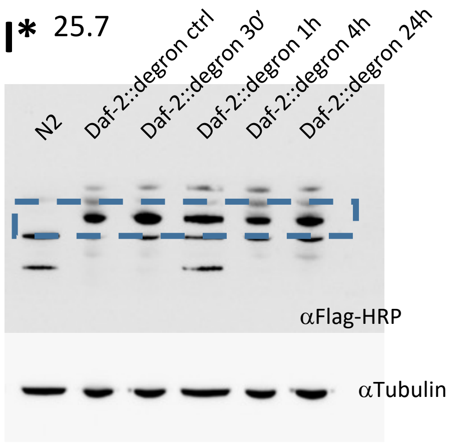

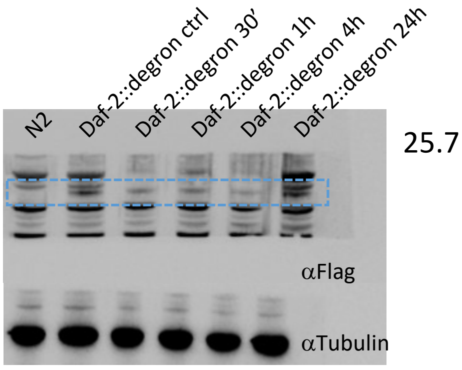

Samples boiled at 95°C

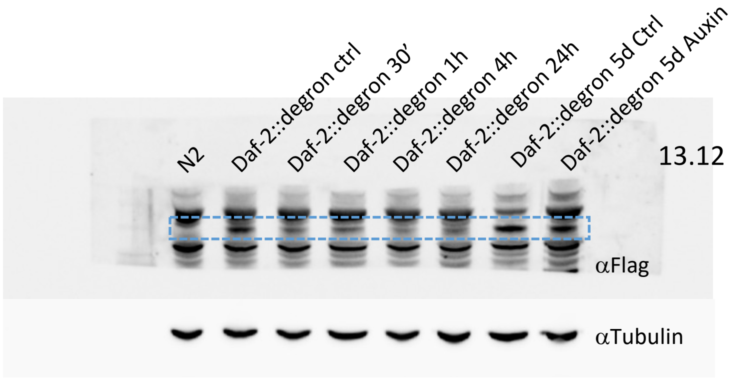

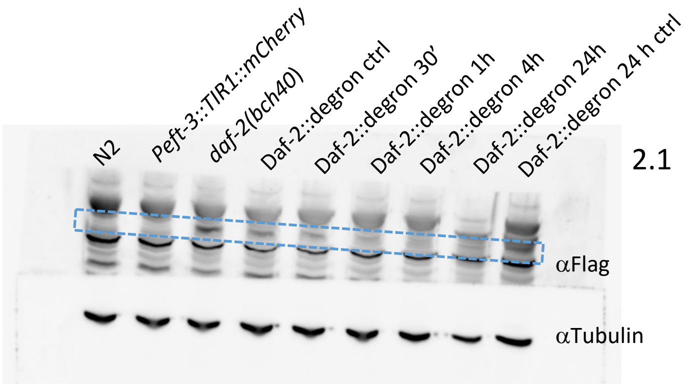

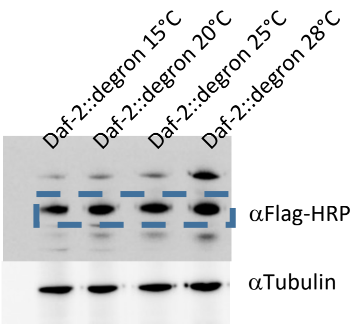

Supplement: Source data 2. — (A-I) Original uncropped western blots. The numbers next to the blot correspond to the trial date. (A–I)* Fully labeled original uncropped western blots. The numbers next to the blot correspond to the trial date. Each relevant band is labeled with the Caenorhabditis elegans strains and treatment conditions. The light blue dotted line corresponds to the cropped area. Additional information about the experimental conditions or to which figure or quantification the blot corresponds is indicated below the blot. [file elife-71335-supp5.pdf]
