# Supplementary material for: End-of-life targeted degradation of DAF-2 insulin/IGF-1 receptor promotes longevity free from growth-related pathologies
Source: eLife. 2021 Sep 10;10:e71335. doi: 10.7554/eLife.71335 (PMC8492056; doi:10.7554/eLife.71335)
Supplement: Source data 3. — Additional repeats and different experimental settings (indicated on the left of each row) for the oxidative stress assays using 5 mM sodium arsenite. Raw data and statistics are in Source data 1. [file elife-71335-supp6.pdf]

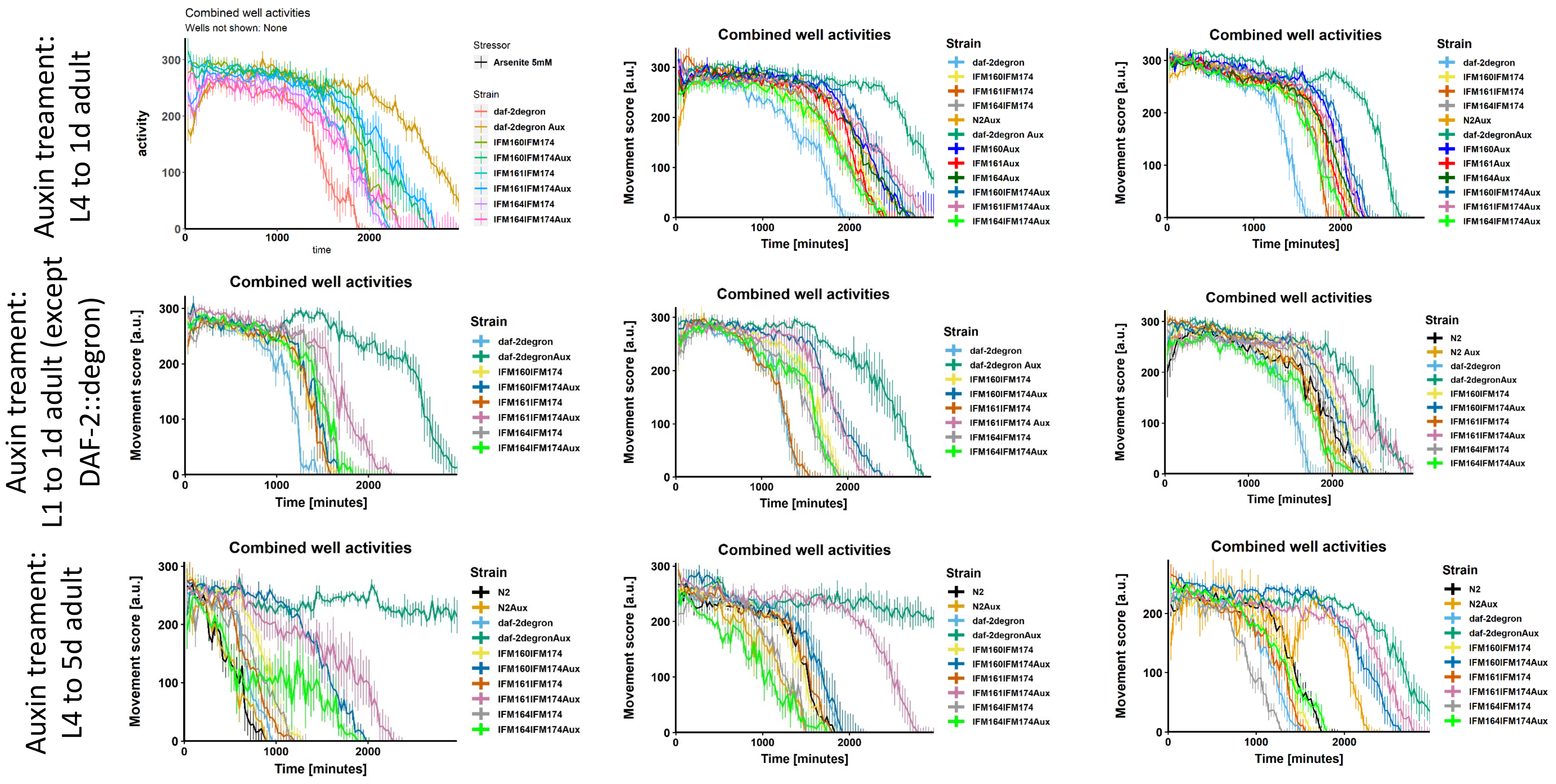
Auxin treament: L4 to 1d adult
Auxin treament: L1 to 1d adult (except DAF-2::degron)
Auxin treament: L4 to 5d adult
Combined well activities
Wells not shown: None
activity
time
Stressor
Arsenite 5mM
Strain
daf-2degron
daf-2degron Aux
IFM160IFM174
IFM160IFM174Aux
IFM161IFM174
IFM161IFM174Aux
IFM164IFM174
IFM164IFM174Aux
Combined well activities
Movement score [a.u.]
Time [minutes]
Strain
daf-2degron
IFM160IFM174
IFM161IFM174
IFM164IFM174
N2Aux
daf-2degron Aux
IFM160Aux
IFM161Aux
IFM164Aux
IFM160IFM174Aux
IFM161IFM174Aux
IFM164IFM174Aux
Strain
N2
N2 Aux
daf-2degron
daf-2degronAux
IFM160IFM174
IFM160IFM174Aux
IFM161IFM174
IFM161IFM174Aux
IFM164IFM174
IFM164IFM174Aux
